# Tree mode of death and mortality risk factors across Amazon forests

**DOI:** 10.1038/s41467-020-18996-3

**Published:** 2020-11-09

**Authors:** Adriane Esquivel-Muelbert, Oliver L. Phillips, Roel J. W. Brienen, Sophie Fauset, Martin J. P. Sullivan, Timothy R. Baker, Kuo-Jung Chao, Ted R. Feldpausch, Emanuel Gloor, Niro Higuchi, Jeanne Houwing-Duistermaat, Jon Lloyd, Haiyan Liu, Yadvinder Malhi, Beatriz Marimon, Ben Hur Marimon Junior, Abel Monteagudo-Mendoza, Lourens Poorter, Marcos Silveira, Emilio Vilanova Torre, Esteban Alvarez Dávila, Jhon del Aguila Pasquel, Everton Almeida, Patricia Alvarez Loayza, Ana Andrade, Luiz E. O. C. Aragão, Alejandro Araujo-Murakami, Eric Arets, Luzmila Arroyo, Gerardo A. Aymard C., Michel Baisie, Christopher Baraloto, Plínio Barbosa Camargo, Jorcely Barroso, Lilian Blanc, Damien Bonal, Frans Bongers, René Boot, Foster Brown, Benoit Burban, José Luís Camargo, Wendeson Castro, Victor Chama Moscoso, Jerome Chave, James Comiskey, Fernando Cornejo Valverde, Antonio Lola da Costa, Nallaret Davila Cardozo, Anthony Di Fiore, Aurélie Dourdain, Terry Erwin, Gerardo Flores Llampazo, Ima Célia Guimarães Vieira, Rafael Herrera, Eurídice Honorio Coronado, Isau Huamantupa-Chuquimaco, Eliana Jimenez-Rojas, Timothy Killeen, Susan Laurance, William Laurance, Aurora Levesley, Simon L. Lewis, Karina Liana Lisboa Melgaço Ladvocat, Gabriela Lopez-Gonzalez, Thomas Lovejoy, Patrick Meir, Casimiro Mendoza, Paulo Morandi, David Neill, Adriano José Nogueira Lima, Percy Nuñez Vargas, Edmar Almeida de Oliveira, Nadir Pallqui Camacho, Guido Pardo, Julie Peacock, Marielos Peña-Claros, Maria Cristina Peñuela-Mora, Georgia Pickavance, John Pipoly, Nigel Pitman, Adriana Prieto, Thomas A. M. Pugh, Carlos Quesada, Hirma Ramirez-Angulo, Simone Matias de Almeida Reis, Maxime Rejou-Machain, Zorayda Restrepo Correa, Lily Rodriguez Bayona, Agustín Rudas, Rafael Salomão, Julio Serrano, Javier Silva Espejo, Natalino Silva, James Singh, Clement Stahl, Juliana Stropp, Varun Swamy, Joey Talbot, Hans ter Steege, John Terborgh, Raquel Thomas, Marisol Toledo, Armando Torres-Lezama, Luis Valenzuela Gamarra, Geertje van der Heijden, Peter van der Meer, Peter van der Hout, Rodolfo Vasquez Martinez, Simone Aparecida Vieira, Jeanneth Villalobos Cayo, Vincent Vos, Roderick Zagt, Pieter Zuidema, David Galbraith

**Affiliations:** 1 School of Geography, Earth and Enviornmental Sciences, University of Birmingham, Birmingham, UK; 2 School of Geography, University of Leeds, Leeds, UK; 3 Birmingham Institute of Forest Research, University of Birmingham, Birmingham, UK; 4 School of Geography, Earth and Environmental Sciences, University of Plymouth, Plymouth, UK; 5 Department of Natural Sciences, Manchester Metropolitan University, Manchester, UK; 6 International Master Program of Agriculture, National Chung Hsing University, Taichung, Taiwan; 7 Geography, College of Life and Environmental Sciences, University of Exeter, Exeter, UK; 8 Instituto Nacional de Pesquisas da Amazônia, Manaus, Brazil; 9 School of Mathematics, University of Leeds, Leeds, UK; 10 Faculty of Natural Sciences, Department of Life, Imperial College London Sciences, London, UK; 11 Environmental Change Institute, School of Geography and the Environment, University of Oxford, Oxford, UK; 12 UNEMAT – Universidade do Estado de Mato Grosso PPG-Ecologia e Conservação, Campus de Nova Xavantina, Nova Xavantina, MT Brazil; 13 Jardín Botánico de Missouri, Oxapampa, Peru; 14 Forest Ecology and Forest Management Group, Wageningen University and Research, Wageningen, Netherlands; 15 Centro de Ciências Biológicas e da Natureza, Universidade Federal do Acre, Rio Branco, AC Brazil; 16 Instituto de Investigaciones para el Desarrollo Forestal (INDEFOR), Universidad de Los Andes, Mérida, Venezuela; 17 University of California, Berkeley, CA USA; 18 Escuela de Ciencias Agropecuarias y Ambientales, Universidad Nacional Abierta y a Distancia, Boyacá, Colombia; 19 Fundación ConVida, Medellín, Colombia; 20 Instituto de Investigaciones de la Amazonia Peruana, Iquitos, Peru; 21 Instituto de Biodiversidade e Florestas, Universidade Federal do Oeste do Pará, Santarém, Brazil; 22 Center for Tropical Conservation, Nicholas School of the Environment, University in Durham, Durham, NC USA; 23 Projeto Dinâmica Biológica de Fragmentos, Instituto Nacional de Pesquisas da Amazônia Florestais, Manaus, AM Brazil; 24 National Institute for Space Research (INPE), São José dos Campos, SP Brazil; 25 Museo de Historia Natural Noel Kempff Mercado, Universidad Autónoma Gabriel Rene Moreno, Santa Cruz de la Sierra, Bolivia; 26 Wageningen Environmental Research, Wageningen University and Research, Wageningen, Netherlands; 27 Dirección de la Carrera de Biología, Universidad Autónoma Gabriel René Moreno, Santa Cruz de la Sierra, Bolivia; 28 INRAE, UMR EcoFoG, CNRS, Cirad, AgroParisTech, Université des Antilles, Université de Guyane, Kourou, France; 29 Department of Biological Sciences, International Center for Tropical Botany, Florida International University, Miami, FL USA; 30 Centro de Energia Nuclear na Agricultura, Universidade de São Paulo, Piracicaba, Brazil; 31 Universidade Federal do Acre, Campus Floresta, Cruzeiro do Sul, Brazil; 32 UR Forest & Societies, CIRAD, Montpellier, France; 33 Department of Biology, Utrecht, Netherlands; 34 Woods Hole Research Center, Falmouth, MA USA; 35 Laboratório de Botânica e Ecologia Vegetal, Universidade Federal do Acre, Rio Branco, AC Brazil; 36 Laboratoire Evolution et Diversite Biologique, CNRS, Toulouse, France; 37 Inventory and Monitoring Program, National Park Service, Fort Collins, CO USA; 38 Proyecto Castaña, Madre de Dios, Peru; 39 Instituto de Geociências, Faculdade de Meteorologia, Universidade Federal do Para, Belém, Brazil; 40 Department of Anthropology and Primate Molecular Ecology and Evolution Laboratory, University of Texas, Austin, TX USA; 41 National Museum of Natural History, Smithsonian Institute, Washington, DC USA; 42 Universidad Nacional Jorge Basadre de Grohmann, Tacna, Peru; 43 Museu Paraense Emílio Goeldi, Belém, Brazil; 44 Instituto Venezolano de Investigaciones Científicas (IVIC), Caracas, Venezuela; 45 IIAMA, Universitat Politécnica de València, València, Spain; 46 Universidad Nacional de San Antonio Abad del Cusco, Cusco, Peru; 47 Instituto Amazónico de Investigaciones Imani, Universidad Nacional de Colombia Sede Amazonia, Leticia, Colombia; 48 Agteca, Santa Cruz, Bolivia; 49 College of Science and Engineering, James Cook University, Cairns, QLD Australia; 50 Department of Geography, University College London, London, UK; 51 Environmental Science and Policy, George Mason University, Fairfax, VA USA; 52 Research School of Biology, Australian National University, Canberra, ACT Australia; 53 School of Geosciences, University of Edinburgh, Edinburgh, UK; 54 Escuela de Ciencias Forestales, Unidad Académica del Trópico, Universidad Mayor de San Simón, Cochabamba, Bolivia; 55 Facultad de Ingeniería Ambiental, Universidad Estatal Amazónica, Puyo, Ecuador; 56 Universidad Nacional de San Antonio Abad del Cusco, Cusco, Perú; 57 Universidad Autónoma del Beni José Ballivián, Trinidad, Bolivia; 58 Universidad Regional Amazónica Ikiam, Ikiam, Ecuador; 59 Broward County Parks Recreation, Oakland Park, FL USA; 60 Keller Science Action Center, Field Museum, Chicago, IL USA; 61 Instituto de Ciencias Naturales, Universidad Nacional de Colombia, Bogotá, Colombia; 62 Institute of Research for Forestry Development (INDEFOR), Universidad de los Andes, Mérida, Venezuela; 63 Socioecosistemas y Cambio Climatico, Fundacion Con Vida, Medellín, Colombia; 64 Centro de Conservacion, Investigacion y Manejo de Areas Naturales, CIMA Cordillera Azul, Lima, Peru; 65 Universidade Federal Rural da Amazônia, Belém, Brazil; 66 Departamento de Biología, Universidad de La Serena, La Serena, Chile; 67 Guyana Forestry Commission, Georgetown, Guyana; 68 Federal University of Alagoas, Maceió, Brazil; 69 Institute for Conservation Research, Escondido, CA USA; 70 Institute for Transport Studies, University of Leeds, Leeds, UK; 71 Biodiversity Dynamics, Naturalis Biodiversity Center, Leiden, The Netherlands; 72 Systems Ecology, Free University, De Boelelaan 1087, Amsterdam, Netherlands; 73 Department of Biology, University of Florida, Gainesville, FL USA; 74 Iwokrama International Centre for Rainforest Conservation and Development, Georgetown, Guyana; 75 Universidad de los Andes, Mérida, Venezuela; 76 School of Geography, University of Nottingham, Nottingham, UK; 77 Van Hall Larenstein University of Applied Sciences, Leeuwarden, Netherlands; 78 Van der Hoult Forestry Consulting, Leeuwarden, The Netherlands; 79 Núcleo de Estudos e Pesquisas Ambientais – Universidade Estadual de Campinas, Campinas, Brazil; 80 Herbario del Sur de Bolivia, Universidad de San Francisco Xavier de Chuquisaca, Sucre, Bolivia; 81 Tropenbos International, Wageningen, Netherlands

**Keywords:** Forest ecology, Tropical ecology

## Abstract

The carbon sink capacity of tropical forests is substantially affected by tree mortality. However, the main drivers of tropical tree death remain largely unknown. Here we present a pan-Amazonian assessment of how and why trees die, analysing over 120,000 trees representing > 3800 species from 189 long-term RAINFOR forest plots. While tree mortality rates vary greatly Amazon-wide, on average trees are as likely to die standing as they are broken or uprooted—modes of death with different ecological consequences. Species-level growth rate is the single most important predictor of tree death in Amazonia, with faster-growing species being at higher risk. Within species, however, the slowest-growing trees are at greatest risk while the effect of tree size varies across the basin. In the driest Amazonian region species-level bioclimatic distributional patterns also predict the risk of death, suggesting that these forests are experiencing climatic conditions beyond their adaptative limits. These results provide not only a holistic pan-Amazonian picture of tree death but large-scale evidence for the overarching importance of the growth–survival trade-off in driving tropical tree mortality.

## Abstract (web summary)

Tree mortality has been shown to be the dominant control on carbon storage in Amazon forests, but little is known of how and why Amazon forest trees die. Here the authors analyse a large Amazon-wide dataset, finding that fast-growing species face greater mortality risk, but that slower-growing individuals within a species are more likely to die, regardless of size.

## Introduction

Tropical forests are key components of the global carbon cycle, and none more so than Amazonia, which stores 150–200 Pg of carbon^1^ and accounts for ~12% of the terrestrial carbon sink^2,3^. Mortality, rather than productivity, controls the spatial distribution of carbon storage across the Basin^4^ and strongly impacts the variation in carbon sink capacity over time^2^. Despite the great significance of tree death to this ecosystem, the contribution of different mechanisms to tree mortality across Amazonia remains unclear. More generally, the poor understanding of risk factors behind tropical tree mortality limits our ability to realistically represent this process in Earth-System models, hampering robust projections of the carbon cycle under future climate scenarios^5,6^.

Tree mortality arises from the interaction of characteristics of the species and the tree with the environment, resulting in physiological failure or structural damage leading to death^7,8^. Physiological failure may be caused by senescence, stress-related (i.e. light competition, moisture stress, pathogen attack) loss of physiological vigour^9^ or by the impairment of water transport as a consequence of hydraulic failure^10,11^. Trees that die from physiology-related causes tend to die standing. Structural failure happens as a consequence of storms and treefalls, leading to stem breakage or uprooting^12^. However, tree death may involve the interaction of several processes. For instance, long-term physiological stress can make trees more vulnerable to ultimately dying from structural failure^8^. Nevertheless, direct observations of the exact processes and conditions that cause tree death are extremely rare (but see refs. ^13,14^), making information from standardised, long-term forest monitoring plots the principal means we have to derive large-scale geographical patterns and differentiate among the potential drivers of tree mortality. In plots, the inferred mode of death (standing vs. broken or uprooted) can be used to provide the basis for understanding the causes of death.

We expect the spatial patterns of the causes of death to be related to the regional variations in climate^15^, forest structure and dynamics^4,12^ present across the Amazon. Previous studies show structural failure to dominate mortality events in the fertile Western region, where trees adopt a more acquisitive strategy, investing more in growth and less in wood structure^12^. Across the Amazon Basin there is a strong gradient of precipitation seasonality, ranging from extremely wet conditions with high rainfall across the entire year in the Northwest to a markedly seasonal climate with a prolonged (up to 7 months) dry season in the South^15^. Death by physiological failure is expected to be greater in drier regions and where the proportion of standing dead trees is higher.

Attributes of individual trees, such as size, are expected to influence the likelihood of tree mortality and provide inference as to the cause of death. For example, mortality by hydraulic failure, observed during extreme drought events, has been shown to disproportionately affect larger trees^10,16–18^. Taller trees with large crowns are also more likely to be struck by lightning^19^. Light competition, on the other hand, is expected to kill mostly small trees, as these tend to experience low light availability and thus be closer to their light compensation point, where they may struggle to fix enough carbon to maintain basic functions^7,20^. Stress conditions, such as a shortage of light or water, may lead to reduced stem growth rate, and ultimately tree death^9,21,22^. Thus, the relative stem diameter growth rate of an individual allows us to infer whether a tree has died from physiological stress. While tree size has been shown to predict tree death^23,24^, recent studies have emphasised the importance of individual growth rate as a mortality risk factor^19^. However, the combined influence of tree size and growth on mortality has only been evaluated for a few sites in the tropics^9^, hindering efforts to understand their general importance as predictors of tree death.

The forensic exercise required to assess the causes of tree mortality is particularly complex in extremely diverse Amazon forests, home to ca. 15,000 tree species^25,26^. These are expected to vary greatly in their baseline mortality rates and tolerance to different potential causes of death^19,24^. The mortality rate of a given species is expected to be predicted by its mean growth rate, reflecting a life-history trade-off between growth and survival^19,27^. Fast-growing taxa tend to have low investment in wood structure, thus being more susceptible to mechanical damage, which leads to shorter life cycles^28^. Meanwhile, taxa with lower growth rates tend to invest more in defence and structure, have high wood density and are expected to have lower mortality rates^29^. Despite theoretical expectations, strong evidence for this trade-off has only been found for saplings and juvenile trees that experience a larger spectrum of light conditions^24,28,30^ but not for adult trees^24,28^. However, the growth-survival trade-off has only really been assessed within single sites and never across large geographical areas. Tolerance to water stress also varies greatly across species, with drought resistance being an important driver of the diversity and distribution of Amazon tree species^31,32^ and is further expected to influence the likelihood of tree death^32,33^.

Here, we analyse >30 years of records from 189 long-term forest plots from the RAINFOR network, including 124,571 trees (≥10 cm of diameter at breast height) and 23,683 tree deaths distributed across Amazonia to provide a biome-scale spatial assessment of mode of tree death. Using a Cox proportional hazard approach, we analyse the risk of death related to characteristics of the individual tree (size and growth prior to death) and species traits (species mean growth rate, maximum stem diameter, wood density and drought tolerance—proxied by biogeographic water-deficit affiliation (WDA)^31^), providing the most comprehensive assessment of the risk factors of tree mortality across Earth’s largest tropical forest domain. Our analyses show the influence of the growth-survival trade-off within adult trees defining large-scale tree mortality patterns and highlight the spatial variation in mortality risk factors across the Amazon basin.

## Results

### Tree mortality rates and mode of death in Amazonia

**Fig. 1:**
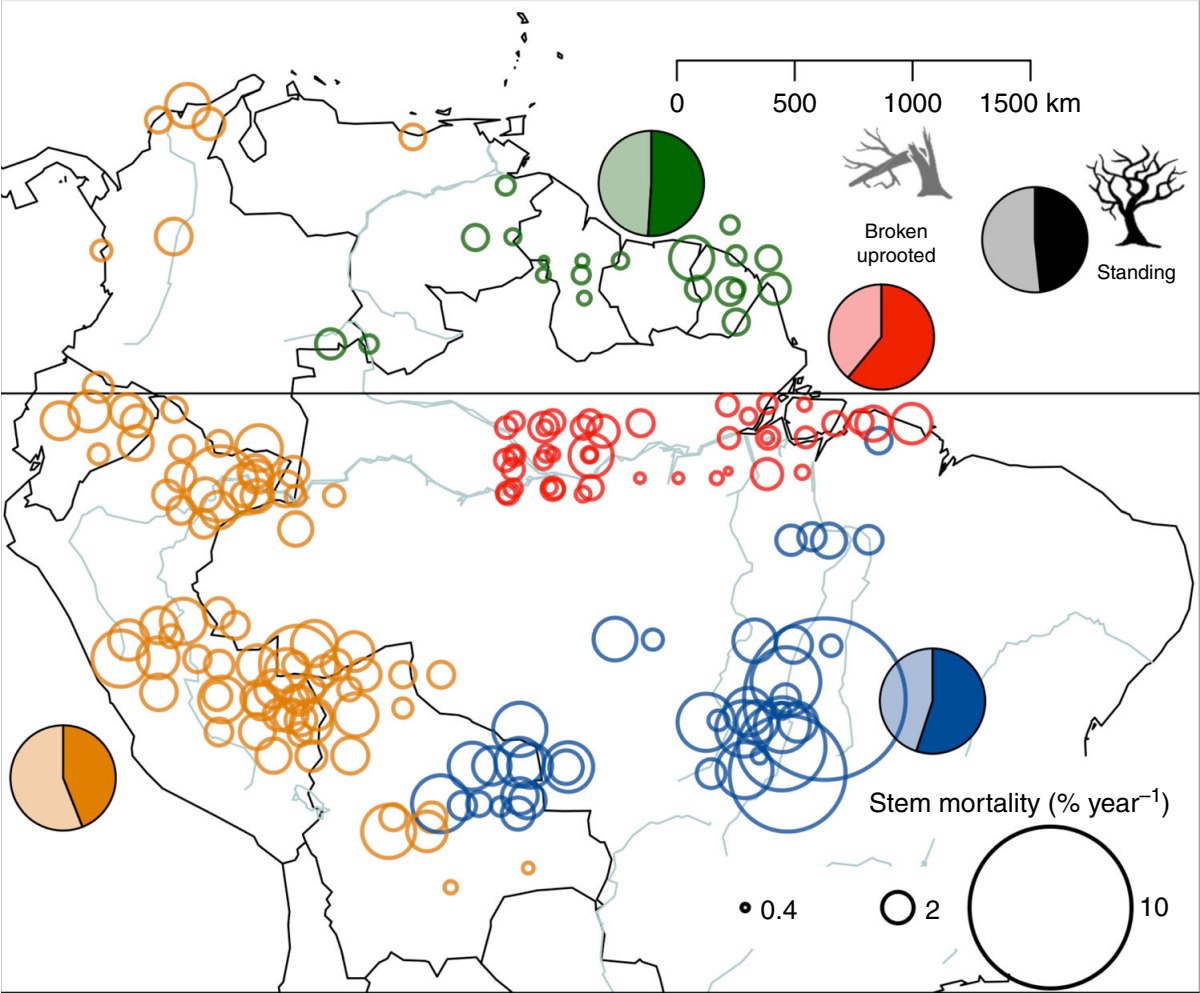
Tree mortality rates and mode of death across Amazonia and adjacent lowland forests. Circles show the mean mortality rate across the entire time series available for each plot (% year^−1^). Pie charts show the proportion of dead trees found standing (darker shading) and broken/uprooted (paler shading). Different colours represent the four geological regions: Northern (green), East-Central (red), Western (yellow) and Southern (blue). Mortality rates per plot were calculated as the mean value across all censuses weighted by the census-interval length.

Mortality rates vary significantly across the Amazon (Fig. 1), being consistently greater in the Western (2.2% year^−1^ [95% confidence intervals (CIs) 2.0–2.3% year^−1^]) and Southern regions (2.8% year^−1^ [2.4–3.4% year^−1^]) than in the much less-dynamic Northern (1.3% year^−1^ [1.2–1.4% year^−1^]) and East-Central regions (1.4% year^−1^ [1.2–1.6% year^−1^]). At the pan-Amazonian scale, trees that were found broken or uprooted, likely to have died as a consequence of structural failure of the stem or roots (often caused by windstorms), represented 51.2% (48–54%) of all Amazon tree death. This proportion is indistinguishable from that of standing dead trees (48.4% [45–52%]) across the basin, in spite of the very different mechanisms involved.

**Fig. 2:**
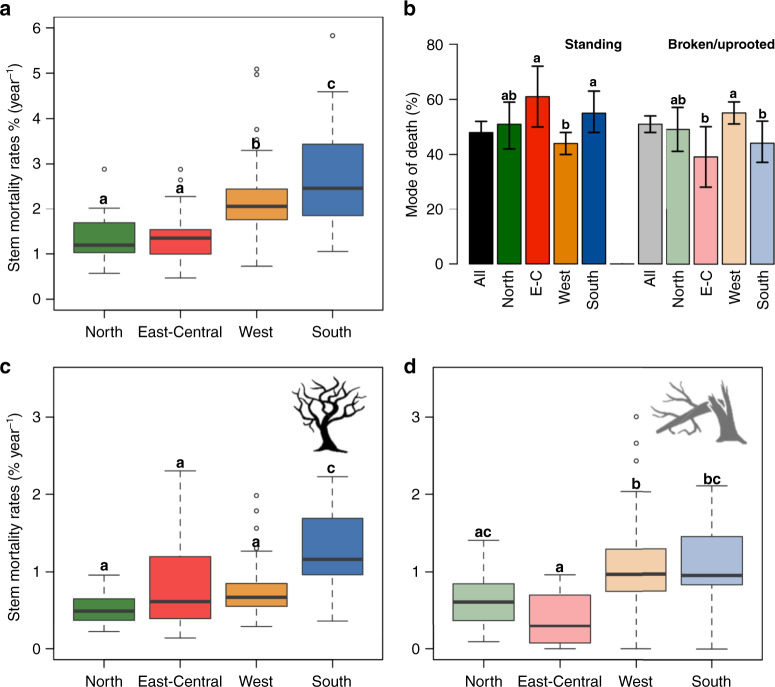
Tree mortality rates in Amazonia. **a** Stem mortality rates per region. **b** Mean proportions and 95% confidence intervals (error bars) of dead trees found standing or broken/uprooted (faded colours). **c** Stem mortality rates for trees that died standing. **d** Stem mortality rates for trees that died broken or uprooted. Different colours represent the four Amazonian geological regions: Northern (green), East-Central (red), Western (yellow) and Southern (blue). Mortality rates per plot were calculated as the mean value per plot across all censuses weighted by the census-interval length. In **a**, **c** and **d**, boxplots show the median, 25th and 75th quantile and whiskers represent 5th and 95th quantile or mortality rates across plots. Letters in **a**–**d** show the results from post hoc Tukey’s tests comparing the proportions and rates among the different regions. Note that in **b** comparisons are independent for standing and for broken/uprooted dead trees. The proportion in **b** and the mortality rates in **c**, **d** were calculated based on 125 plots where at least 50% of dead trees and at least 5 trees had their mode of death registered.

As expected, where mortality rates were higher, the absolute rates of both broken/uprooted death and standing death also tended to be higher (Fig. 2, Appendix S1). However, we did not observe a consistent link between regional patterns in mortality rates and the relative importance of different modes of death (Figs. 1 and 2). The proportion of trees found either broken/uprooted or standing after death did differ between the highly dynamic Western region, where most trees die broken/uprooted (55%, [51–59%]), and East-Central Amazonia, where mortality rates are low and broken/uprooted trees accounted only for 39% (28–50%) of tree death. However, in the most dynamic forests of Southern Amazonia, broken/uprooted trees contributed to only 44% (37–52%) of tree death. In the least-dynamic Northern region, the proportion of broken/uprooted (49%, [41–57%]) and standing death (51%, [42–59%]) were equivalent and did not differ significantly from the much more dynamic Southern region.

### Factors influencing risk of tree death

Tree mortality risk across the Amazon depends both on the characteristics of the individual tree, and on its species-level traits (Table 1). Models explaining tree death with both tree-level variables and species traits performed better than models with either group of risk factors alone (Table 1). But the condition of tree and species is not equal: models with only species traits (∆AIC = 497) predict mortality better than models containing only tree-level attributes (∆AIC = 3283) (Table 1).

**Table 1:** Comparison between different Cox proportional hazard models predicting tree mortality across Amazonian forests.

Tree-level coefficients	Species-level coefficients	∆AIC	Model description
Rel. growth + *D* + *D*^2^	Max *D* + mean growth + WD + WDA	0	Full model
Rel. growth + *D* + *D*^2^	Max *D* + mean growth + WD	0.4	Excluding WDA
Rel. growth + *D*	Max *D* + mean growth + WD + WDA	132	Linear relationship with size
Rel. growth + *D* + *D*^2^	Max *D* + mean growth + WDA	139	Excluding WD
Rel. growth	Max *D* + mean growth + WD + WDA	226	Excluding stem size
*D* + *D*^2^	Max *D* + mean growth + WD + WDA	260	Excluding stem relative growth
	Max *D* + mean growth + WD + WDA	497	Species-level risk factors only
Rel. growth + *D* + *D*^2^	Mean growth + WD + WDA	1330	Excluding species max size
Rel. growth + *D* + *D*^2^	Max *D* + WD + WDA	1734	Excluding species mean growth
	Mean growth	2652	Species mean growth only
Rel. growth + *D* + *D*^2^		3283	Tree-level risk factors only
Rel. growth		3591	Relative growth only
		3646	Null model

Models vary according to risk factors considered, including tree-level characteristics: size, represented by tree diameter (*D*) and relative stem diameter growth rates (rel. growth) and species traits: maximum stem diameter size (max *D*), mean stem diameter growth rate (mean growth), wood density (WD) and drought tolerance represented as water-deficit affiliation^33^ (WDA). The importance of each risk factor is represented by comparing models based on the difference in Akaike’s Information Criterion (ΔAIC). The model with the lowest AIC is the one that contains the best combination of variables and is used as the reference for model comparison. Models are considered different when ΔAIC is >2. The full model was the best model after comparison using the *stepAIC* R function.

**Fig. 3:**
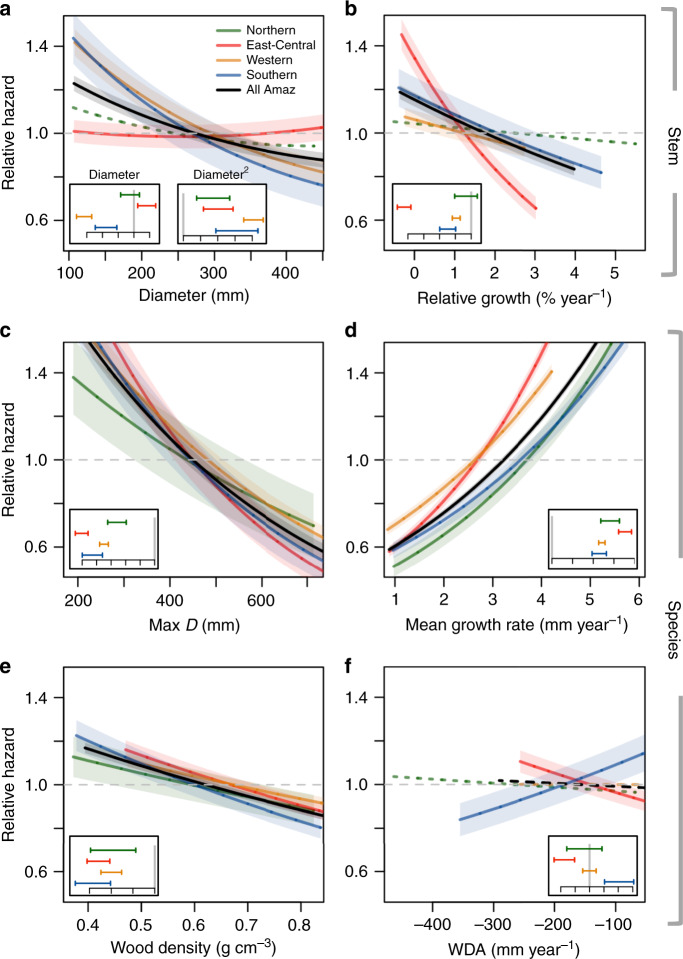
Risk factors of Amazon tree death. Cox proportional model outputs for the risk factors associated with the tree-level characteristics: **a** stem diameter size and **b** relative stem growth rates; and for species traits: **c** maximum stem diameter size (max *D*), **d** mean stem diameter growth rate, **e** wood density and **f** drought tolerance represented as water-deficit affiliation, WDA. The WDA values were obtained from a previous study, calculated as the mean of maximum cumulative water-deficit (mm year^−1^) where the species occurred weighted by its abundance^33^. More negative values indicate that the species occur under drier conditions, and had greater survival in drought experiments^34^. Inserts show the coefficients and respective 95% confidence intervals for each variable in every region. Black lines show the models for the entire basin and different colours represent the four Amazonian geological regions: Northern (green), East-Central (red), Western (yellow) and Southern (blue). Shaded area represents the standard error for each coefficient and dotted lines represent non-significant risk factors. Note that for visualisation purposes, we restricted the figure to the 95th quantile of the distribution of each variable.

Species mean growth rate was the best predictor of tree death, accounting for the highest individual *χ*^2^ in all regions and being the single risk factor whose removal from the full model resulted in the highest ∆AIC (1734) (Tables 1 and 2). In all regions, fast-growing species were at higher risk. All predictors except WDA were found to be important risk factors in the pan-Amazonian analysis, with smaller and light wooded species having higher mortality rates (Table 2 and Fig. 3c–e).

**Table 2:** Parameters from the best Cox proportional hazard model of Amazon tree mortality.

		*D*	*D*^2^	Rel. growth	Max *D*	Mean growth	WD	WDA
All Amazonia	Coef (SE)	**−28 (3)**	**26 (2)**	**−0.08 (0.01)**	**−0.002 (0.0001)**	**0.2 (0.005)**	**−0.7 (0.06)**	−1 × 10^−4^ (1 × 10^−4^)
(*n* = 11,6431; *d* = 21,272)	*χ*^2^	101	216	233	1190	2126	141	2
Northern Amazonia	Coef (SE)	−2.5 (3)	**8.6 (2.4)**	−0.02 (0.02)	**−0.001 (0.0002)**	**0.2 (0.02)**	**−0.5 (0.2)**	−2 × 10^−4^ (3 × 10^−4^)
(*n* = 17,585; *d* = 2307)	*χ*^2^	0.6	13	1	64	188	9	0.3
East-Central Amazonia	Coef (SE)	**8 (3)**	**10 (2)**	**−0.2 (0.01)**	**−0.003 (0.0001)**	**0.3 (0.01)**	**−0.8 (0.1)**	**−9** × **10**^**−4**^ **(2** × **10**^**−4**^**)**
(*n* = 39,281; *d* = 6077)	*χ*^2^	8	21	289	500	668	45	23
Western Amazonia	Coef (SE)	**−32 (2)**	**20 (1.4)**	**−0.05 (0.01)**	**−0.002 (0.0001)**	**0.22 (0.006)**	**−0.52 (0.08)**	−1 × 10^−5^ (1 × 10^−4^)
(*n* = 45,432; *d* = 10,603)	*χ*^2^	166	199	54	511	1154	41	0
Southern Amazonia	Coef (SE)	**−18 (4)**	**16 (3)**	**−0.08 (0.01)**	**−0.002 (0.0002)**	**0.2 (0.01)**	**−0.9 (0.2)**	**1** × **10**^**−3**^ **(3** × **10**^**−4**^**)**
(*n* = 14,133; *d* = 2285)	*χ*^2^	26	25	31	145	205	21	16

Coefficients and standard errors, in brackets, and *χ*^2^ for each risk factor shown for the model using data from the whole Amazon (All Amazon) and for models describing tree mortality in each of the four Amazon geological regions. Risk factors include characteristics from the trees: size, represented by tree diameter (*D*) and relative stem diameter growth rates (rel. growth); and species traits: maximum stem diameter size (max *D*), mean stem diameter growth rate (mean growth), wood density (WD) and drought tolerance represented as water-deficit affiliation^33^ (WDA). In bold are the coefficients that significantly differ from zero considering *α* = 0.05. Number of trees included in the analysis (*n*) and number of dead trees (*d*) are shown for each region.

When considering tree-level predictors, relative growth rates were a significant risk factor for Amazonian trees (Fig. 3 and Table 2), greatly enhancing the performance of mortality models (∆AIC = 260 when tree-level growth was excluded from the full model, Table 1). Tree size was also an important risk factor for tree death; however, it was less so than growth rate (∆AIC = 226, Table 1).

Although risk factors associated with mortality were generally consistent among the different Amazonian regions, we observed some spatial variation in the coefficients associated with specific risks (Fig. 3 and Table 2). For instance, in Southern Amazonia, the driest of all regions, species tolerance to drought was an important predictor of tree mortality, with wet-affiliated species being at greater risk (Fig. 3f). In East-Central Amazonia, the only region where standing death was more prevalent than broken/uprooted death (Appendix S1 and Fig. 2), the risk associated with tree-level relative growth rate was greater than in any other region (Fig. 3b and Table 2). In Western and Southern Amazon, smaller trees were at greater mortality risk, while in East-Central Amazonia, larger trees were more at risk (Fig. 3a and Table 2).

We repeated our pan-Amazonian risk analysis independently for trees that died standing and for those that died uprooted/broken. Again, as for the general model, species attributes were more important than tree-level factors for both modes of death (Table S4). However, we found differences in the relative importance of specific risk factors for different modes of death (Tables S4 and S5), with slower-growing trees tending to be at greater risk of standing death than of being broken/uprooted (Figure S2 and Table S5).

## Discussion

We provide the most comprehensive and geographically distributed assessment of Neotropical tree mortality yet attempted. Previous studies of the mode of tree death in Amazon forests have been highly localised in nature (e.g. refs. ^13,34,35^) or restricted to a small number of plots (e.g. ref. ^12^). We show that catastrophic structural damage is likely to be a common cause of mortality across the Amazon, with 51.2% (SE = 48–54%) of dead trees being found broken or uprooted. It has been suggested that the proportion of trees that die from structural failure should be related to overall mortality rates^12,36^. We find little evidence for such a relationship: while the proportion of trees dying broken/uprooted does dominate in the dynamic forests of Western Amazonia, in both the most dynamic region of the Amazon—the South—and the least—the North—broken/uprooted and standing death were in similar proportion to each other (Fig. 1). These results thus demonstrate that spatial variation in mortality rates cannot be explained simply based on a physiological (standing) rate, which is incremented by a spatially varying risk of mortality by structural failure, as previously proposed^36^. Instead, our results suggest that competition and other drivers of physiological failure also show large spatial variation.

We found species life-history strategies to be more important than the characteristics of the individual trees for predicting tree mortality across Amazonia (Tables 1 and 2), consistent with previous local studies^19^. In particular, we show that a great part of the variation in the hazard rates is explained by the species mean growth rates (Table 1). This result provides strong empirical support for the growth-longevity trade-off hypothesis across tropical species^29^, showing that this trade-off is also pervasive across adult trees and consistent across forests with distinct species composition and under different climatic and edaphic characteristics^36^.

The growth of individual trees was a fundamental predictor of mortality across all Amazonian regions, indicating that trees often undergo a period of slow growth prior to death (Tables 1 and 2). Despite the overall importance of tree growth across the whole Basin, the risk factor associated with it is greater in East-Central Amazonia, where most dead trees are found standing (Figs. 1 and 3). Interestingly, individual growth was an important predictor of mortality for trees that died broken or uprooted, as well as those that died standing (Table S5), suggesting that some degree of physiological failure may increase the susceptibility of these trees to being broken or uprooted (Table S5 and Figure S2).

Observational studies often focus on tree size as a predictor of death^16,17,23,24^. Here, although both the linear and the U-shaped risk factor related to tree size were significant for the model at the Basin level, the effect of size was not consistent across the different regions. The decreasing mortality risk with size in Western and Southern Amazon (Fig. 3a and Table 2) can be understood in terms of the dominance of broken/uprooted as a mode of death. When a tree is broken or uprooted, it is likely to kill several smaller ones, resulting in greater risk for smaller trees. In addition to this collateral death, in the drier Southern region belowground competition for water may also contribute to the greater death of smaller trees. The opposite effect is observed in East-Central Amazonia, where broken/uprooted death is rare and larger trees were at greater risk (Table 2).

Despite evidence for occasional widespread acute events of large drought-induced mortality^14,37–39^, our results suggest that drought is a significant driver of tree death only in the dry southern fringes of the basin^15^ where species water-deficit affiliation had a significant role in predicting mortality (Fig. 3 and Table 2). This is somewhat surprising as these forests are expected to be relatively adapted to dry conditions when compared to other Amazon forests. This result could be an outcome of drought-adapted species having longer lifespans when compared to drought-vulnerable ones. However, this seems not to be the case in our data as we find no relationship between the species growth rates and their drought affiliation for Southern Amazonia (*R*^2^ = 0.0001, *P* value = 0.1). Thus, the selective mortality of the most vulnerable species indicates that these communities are already experiencing climatic extremes that go beyond the conditions to which these species are adapted^40,41^. Surprisingly, drought-tolerant species were at greater risk of death in East-Central Amazonia. We interpret this to be a potential consequence of a trade-off between flood and drought resistance. Mortality here has been previously related to wet climate anomalies^14^ and this region suffered flooding caused by the extreme 1989 wet season, known to have increased tree mortality rates of particular species^42^.

Our results also have major implications for modelling tropical forest dynamics. Fully capturing the dynamics of tropical forests in vegetation models, including those in Earth-System models, will require explicit computation of tree demography, alongside plant functional descriptions that include tree longevity strategies. Together, the species traits and tree-level predictors identified here can provide a robust empirical underpinning for simulating tree mortality in the Amazon. The empirical relationships found here can be directly incorporated into individual-based size-structured vegetation models, such as done by Fauset et al.^43^. The linkage between mortality probability and individual relative growth can also be readily incorporated into the size-cohort-based vegetation models, which are becoming increasingly widespread^6^, replacing widely applied theoretical approaches, which are hard to parameterise in practice^44^. However, even models without a full cohort structure can still benefit substantially from the relationships identified here (Table 1). Such implementations in models will also benefit from further work to link these equations to environmental variables.

In summary, we show that the risk factors related to tree death vary across the different Amazonian regions. Species traits predicted tree death better than the tree-level characteristics, indicating that changes in species composition across these forests^33^ are likely to alter their baseline mortality rates. Climate also contributes to the spatial variation in risk, with species drought vulnerability significantly predicting death in the dry fringes of the Amazon. Thus, forests at the limits of the biome are potentially experiencing climatic conditions beyond those to which they are optimally adapted. Future work should focus on the temporal analysis of risk factors shown here to gain insights into the potential drivers of increasing tree mortality documented across Amazon forests^2^. Together, our results change the current understanding of the macroecological patterns of tree death in the tropics and can help predict the future dynamics of the largest tropical forest on Earth.

## Methods

### Forest inventory data

We investigated tree mortality in 189 long-term forest inventory plots across the Amazon basin as part of the RAINFOR^45^ network, accessed via the ForestPlots.net repository^46,47^. All plots analysed are located in lowland (<1000 m.a.s.l.), *terra firme*, intact forest and were monitored regularly—we did not include in the analyses plots in which the difference between census intervals was >10 years. Plots smaller than 0.5 ha were excluded, or else joined together when <1 km apart. The average census interval is 2.8 years (95% CI = 2.7, 2.9) and the average plot size is 1.23 ha (95% CI = 1.1, 1.37) with a total area of 331.05 ha.

Plot monitoring followed a standard protocol^48^ for which full details can be found elsewhere^2^. In brief, all trees and palms that have a stem diameter at 1.3 m (or above buttresses) of ≥10 cm are measured, tagged and identified, when possible, to the species level. In every census, when the plot is revisited, the living trees are measured, the new recruits that attain stem diameter ≥10 cm are tagged and measured, and notes are taken about the dead trees. Lianas and nonwoody arborescent individuals from the families Strelitziaceae and Cyatheaceae were excluded from these analyses.

### Mortality rates

Plot-level mortality rates were calculated for the 189 plots as the mean mortality rates across all censuses, weighted by the census-interval length between two consecutive censuses. Tree mortality rates in % year^−1^ for each census were calculated as^49^

1 $$m = \left( {1 - \left( {\frac{{N_{t1}}}{{N_{t0}}}} \right)^{\frac{1}{T}}} \right) \times 100,$$

where *N*_*t*1_ is the number of individuals that survived the census interval, *N*_*t*0_ the initial number of individuals and *T* the time span between two consecutive censuses. To provide a better understanding of the spatial drivers of mortality, mortality rates were also calculated for four Amazon regions (Northern, East-Central, Western and Southern Amazonia) that differ strongly in geological age and soil substrate^50^. Mortality rates for the different regions were compared using post hoc Tukey’s test by applying the function *TukeyHSD* from the R package *stats*^51^.

Basin- and region-level mortality rates were estimated as the bootstrapped mean and 95% CI of the mortality rates weighted by the area of the plot calculated from 10,000 weighted means of randomly resampled values of plot‐level mortality rates across all plots.

Dead trees were diagnosed as having died standing or non-standing (broken or uprooted) following a standardised protocol for assessing the mode of death based on an analysis of the tree when it is found dead^12,48^. This information allowed us to assess the proportion of trees within different modes of death and to calculate mortality rates for each of them. These rates were calculated using Eq. 1, but in this case *N*_*t*1_ is the number of individuals that did not die either standing or broken/uprooted. This analysis included 125 plots where the mode of death was recorded following a standardised protocol^48^ for at least 50% of the dead trees and at least 5 individuals. This represents a total of 16,599 dead trees assessed for mode of death.

Depending on the length of the census interval, trees that die standing might break. Although the protocol allows for trees that are found broken to be classified as having died standing if there are indications that that was the case^12,48^, the proportion of standing vs. broken trees might depend on the length of the census interval. To correct for this potential bias, we accounted for the census-interval length when calculating the proportion of trees within these two modes of death groups (standing and broken/uprooted). First, we tested the influence of census interval on these proportions by fitting linear models where the plot-level proportion of dead trees in one of these groups (standing and broken/uprooted) (Pmod) is a function of the mean census-interval length across all censuses in a given plot ($\overline {{\mathrm{CIL}}}$):

2 $${\mathrm{Pmod}} = \beta _0 + \beta _1\overline {{\mathrm{CIL}}} + \varepsilon.$$

This approach allowed us to determine that the proportion of broken/uprooted dead trees increases by 4% year^−1^ (*R*^2^ = 0.12, *p* value <0.01). In Eq. 2 we centred the $\overline {{\mathrm{CIL}}}$ to have a mean of zero and used the intercept of the model (*β*_0_) as the corrected proportion of trees that died standing or broken/uprooted. Here *β*_0_ represents the proportion of a certain mode of death at the mean $\overline {{\mathrm{CIL}}}$ across all plots.

Subsequently, we estimated the proportions of each mode of death for the different geological regions while accounting for the effect of census-interval length by including it as a covariate in a model of mode of death against region:

3 $${\mathrm{Pmod}} = \beta _0 + \beta _1\overline {\mathrm{CIL}} + \beta _2{\mathrm{region}} + \varepsilon.$$

In Eq. 3 we estimated the regional proportions of each mode of death to be estimated while statistically controlling for the effect of census-interval length. We tested for the differences in the proportions of trees found standing vs. those found broken/uprooted within and among the different Amazonian regions by comparing the 95% CIs around the regional means from Eq. 3, using the function *confint* from the R package *stats*^51^. We further applied a post hoc Tukey’s test comparing the difference in mode of death across Amazonian regions using the function *glht* from the R package *multcomp*^52^.

### Species traits and tree-level information

Species traits (wood density, maximum size, mean growth and climate affiliation) were obtained from previous studies. Wood density data (in g cm^−3^) were obtained using previous studies from measurements in different areas of the Amazon^53^. WDA (in mm) was derived in a previous study using relative abundances across 513 inventory plots distributed along a large water-deficit gradient across the Western Neotropics^31^. WDA has shown to be an important metric of drought vulnerability successfully predicting drought-induced mortality in several drought experiments from different Neotropical forests^32^. Mean growth (in mm year^−1^) was obtained from Coelho de Souza et al.^29^ and maximum stem diameter size (in mm) was estimated by Coelho de Souza et al.^29^ and Esquivel-Muelbert et al.^33^, these previous studies were based on a large number of inventory plots distributed across Amazonia. The maximum size represents the 95th quantile of the distribution of size and growth rates across all individuals of a given species^29,33^. In the cases where species-level traits were missing for species the mean trait value of the genus was used. If the genus information was missing, we used the mean trait value of the family. To those trees belonging to families that had no trait information, we assigned the mean trait value of all individuals of the plot (cf. refs. ^29,54,55^). Species-, genus- and family-level maximum size data were missing for 14%, 6% and 3% of the stems, respectively. For mean growth rates at species, genus and family level, information was missing for 16%, 7% and 3% of the stems (Table S2).

The characteristics of the individual tree considered were its size (diameter, *D*) and relative growth rate (rel. growth), calculated as

4 $${\mathrm{rel}}.\,{\mathrm{growth}} = \frac{{(D_{t1} - D_{t0})/T}}{{D_{t0}}},$$

where *T* is the time span between the antepenultimate (*t*_0_) and the penultimate census (*t*_1_) when the tree was observed in our data. *D*_*t*0_ and *D*_*t*1_ are the diameter in the antepenultimate and the penultimate census, respectively. Palms (Arecaceae) were excluded from the main survival analyses as they do not have horizontal growth. Trees with relative growth rate more negative than −5% year^−1^ (75 in total, 0.06% of the total number of stems) were excluded from the analyses, as such negative stem growth is not biologically possible and likely to be a measurement error.

Size was obtained from the penultimate census in which the tree was recorded (*D*_*t*0_). Previous studies indicate a U-shape relationship between diameter and mortality^23,24^. We tested for this U-shape relationship including diameter (*D*) in our models as a polynomial function:

5 $$f_{{\mathrm{size}}} = \beta _1D + (\beta _2D^2).$$

### Analytical approach

We performed survival analyses to identify the risk of death related to different species traits and the condition of individual trees. We used the Cox proportional hazard model, which estimates the influence of risk factors on the time-to-event response. This model differs from logistic regression as it accounts for the time to event (here time to death) to occur for each individual tree^56^. Our models included risk factors that describe the characteristics of the tree (relative growth rate and tree size) and characteristics of the species (i.e. mean growth rate, maximum diameter, wood density and drought affiliation):

6 $$h(t) = h_0(t) \times {\mathrm{exp}}(X{\prime}\beta + zb),$$

where *h*_0_ is the baseline mortality, *t* is the time for the mortality to happen, **X** is a vector of risk factors (*x*_1_, *x*_2…_), **β** is a vector of the corresponding coefficients, *z* is the random effect, that is, plot, and *b* its corresponding coefficient. We consider plot as a random effect (*z*), as trees are nested within plots and this factor allows us to account for plot characteristics, for example. number of censuses, edaphic and climatic conditions.

Our compilation of species- and tree-level characteristics resulted in seven potential predictors for our analyses. To verify potential collinearity between these variables (Figure S1), we calculated the variance inflation factor (VIF) for the model including all variables (Table S3) using function *vif* from the R package *rms*^57^. As none of the initial predictive variables show high VIF^58^ (i.e. >10) they were all maintained in our analyses.

To select the combination of variables that best predicted mortality, we performed a forward and backward selection on the full model with the risk factors described above. We used the function *stepAIC* from the R package *MASS*^59^ selecting for the model that minimises the Akaike’s information criterion (AIC)^60,61^. Finally, the importance of individual risk factors in describing mortality was tested by comparing the AIC of models with different structures and by comparing the *χ*^2^ associated with each risk factor.

To understand how the causes of mortality vary across the Amazon, the survival analysis described above was repeated for each of the four Amazonian geological regions: Northern, East-Central, Western and Southern. The best model selected for the whole basin was applied to each of the regions allowing comparison for risk factors among them.

To perform the survival analysis, we used data from 158 plots that were monitored three or more times. This included information from 116,431 trees, of which 21,272 died during the monitoring period. This analysis was repeated for trees that died standing and fallen (i.e. broken and uprooted) separately for 68,593 trees and 11,980 deaths (3639 standing, 5409 fallen and 2932 with mode of death not identified) within the 116 plots where this information was available and followed the criteria described here and in the *Forest inventory data* section (results are presented in Appendix S3). All analyses were performed using the R software version 3.5.2^51^. The R package *survival* was used for all survival analyses^62^.

### Reporting summary

Further information on research design is available in the Nature Research Reporting Summary linked to this article.

## Supplementary information

Supplementary Information

Reporting Summary

## Data Availability

The source data underlying the analyses in the main text are available at https://www.forestplots.net/en/publications#data. Source data are provided with this paper.

## Source data

Source Data

## Code availability

The codes and instructions to perform the analyses and generate the figures shown in the main text are available at https://github.com/AEMuelbert/AmazonTreeMortality.git; 10.5281/zenodo.3979106. Data for the analyses are available as a ForestPlots.net data package at 10.5521/forestplots.net/2020_6. Source data are provided with this paper.

## References

[1] Feldpausch TR (2012). Tree height integrated into pantropical forest biomass estimates. Biogeosciences.

[2] Brienen RJW (2015). Long-term decline of the Amazon carbon sink. Nature.

[3] Le Quéré C (2018). Global Carbon Budget 2017. Earth Syst. Sci. Data.

[4] Johnson, M. O. et al. Variation in stem mortality rates determines patterns of above-ground biomass in Amazonian forests: implications for dynamic global vegetation models. *Glob. Change Biol.*10.1111/gcb.13315, 3996–3401 (2016). 10.1111/gcb.13315 PMC6849555 27082541

[5] Friend AD (2014). Carbon residence time dominates uncertainty in terrestrial vegetation responses to future climate and atmospheric CO2. Proc. Natl Acad. Sci. USA.

[6] Fisher RA (2018). Vegetation demographics in Earth System Models: a review of progress and priorities. Glob. Change Biol..

[7] McDowell, N. et al. Drivers and mechanisms of tree mortality in moist tropical forests. *New Phytol*. **219**, 851–869 (2018). 10.1111/nph.15027 29451313

[8] Franklin JF, Shugart HH, Harmon ME (1987). Tree death as an ecological process. Bioscience.

[9] Chao KJ (2008). Growth and wood density predict tree mortality in Amazon forests. J. Ecol..

[10] Rowland L (2015). Death from drought in tropical forests is triggered by hydraulics not carbon starvation. Nature.

[11] McDowell NG (2011). Mechanisms linking drought, hydraulics, carbon metabolism, and vegetation mortality. Plant Physiol..

[12] Chao KJ, Phillips OL, Monteagudo A, Torres-Lezama A, Martinez RV (2009). How do trees die? Mode of death in northern Amazonia. J. Veg. Sci..

[13] Fontes CG, Chambers JQ, Higuchi N (2018). Revealing the causes and temporal distribution of tree mortality in Central Amazonia. Ecol. Manag..

[14] Aleixo I (2019). Amazonian rainforest tree mortality driven by climate and functional traits. Nat. Clim. Change.

[15] Sombroek W (2001). Spatial and temporal patterns of Amazon rainfall—consequences for the planning of agricultural occupation and the protection of primary forests. Ambio.

[16] Bennett AC, McDowell NG, Allen CD, Anderson-Teixeira KJ (2015). Larger trees suffer most during drought in forests worldwide. Nat. Plants.

[17] Phillips OL (2010). Drought-mortality relationships for tropical forests. N. Phytol..

[18] Liu H (2019). Hydraulic traits are coordinated with maximum plant height at the global scale. Sci. Adv..

[19] Gora, E. M. et al. A mechanistic and empirically-supported lightning risk model for forest trees. *J. Ecol*. 10.1111/1365-2745.13404 (2020).

[20] Camac, J. S. et al. Partitioning mortality into growth-dependent and growth-independent hazards across 203 tropical tree species. *Proc. Natl Acad. Sci. USA*10.1073/pnas.1721040115 (2018). 10.1073/pnas.1721040115 PMC6298112 30446609

[21] Cailleret M (2017). A synthesis of radial growth patterns preceding tree mortality. Glob. Change Biol..

[22] Wyckoff PH, Clark JS (2002). The relationship between growth and mortality for seven co-occurring tree species in the southern Appalachian Mountains. J. Ecol..

[23] Lines, E. R., Coomes, D. A. & Purves, D. W. Influences of forest structure, climate and species composition on tree mortality across the Eastern US. *PLoS ONE* **5**, 10.1371/journal.pone.0013212 (2010). 10.1371/journal.pone.0013212 PMC2954149 20967250

[24] Iida Y (2014). Linking functional traits and demographic rates in a subtropical tree community: the importance of size dependency. J. Ecol..

[25] ter Steege H (2013). Hyperdominance in the amazonian tree flora. Science.

[26] ter Steege H (2020). Biased-corrected richness estimates for the Amazonian tree flora. Sci. Rep..

[27] Reich PB (2014). The world-wide ‘fast–slow’ plant economics spectrum: a traits manifesto. J. Ecol..

[28] Wright SJ (2010). Functional traits and the growth–mortality trade-off in tropical trees. Ecology.

[29] Coelho de Souza, F. et al. Evolutionary heritage influences Amazon tree ecology. *Proc. R. Soc. Ser. B* **283**, 10.1098/rspb.2016.1587 (2016). 10.1098/rspb.2016.1587 PMC5204144 27974517

[30] Zhu Y (2018). Density-dependent survival varies with species life-history strategy in a tropical forest. Ecol. Lett..

[31] Esquivel-Muelbert A (2017). Seasonal drought limits tree species across the Neotropics. Ecography.

[32] Esquivel-Muelbert A (2017). Biogeographic distributions of neotropical trees reflect their directly measured drought tolerances. Sci. Rep..

[33] Esquivel-Muelbert A (2019). Compositional response of Amazon forests to climate change. Glob. Change Biol..

[34] de Toledo JJ, Magnusson WE, Castilho CV, Nascimento HEM (2012). Tree mode of death in Central Amazonia: effects of soil and topography on tree mortality associated with storm disturbances. Ecol. Manag..

[35] Gale N, Barfod AS (1999). Canopy tree mode of death in a western Ecuadorian rain forest. J. Trop. Ecol..

[36] Quesada CA (2012). Basin-wide variations in Amazon forest structure and function are mediated by both soils and climate. Biogeosciences.

[37] Feldpausch TR (2016). Amazon forest response to repeated droughts. Glob. Biogeochem. Cycle.

[38] Saatchi S (2013). Persistent effects of a severe drought on Amazonian forest canopy. Proc. Natl Acad. Sci. USA.

[39] Phillips OL (2009). Drought sensitivity of the Amazon rainforest. Science.

[40] Anderegg WRL, Anderegg LDL, Kerr KL, Trugman AT (2019). Widespread drought-induced tree mortality at dry range edges indicates that climate stress exceeds species’ compensating mechanisms. Glob. Change Biol..

[41] Tiwari, R. et al. Photosynthetic quantum efficiency in south-eastern Amazonian trees may be already affected by climate change. *Plant Cell Environ.*10.1111/pce.13770 (2020). 10.1111/pce.13770 32339294

[42] Nelson BW (2005). Pervasive alteration of tree communities in undisturbed Amazonian forests. Biotropica.

[43] Fauset, S. et al. Individual-based modeling of Amazon forests suggests that climate controls productivity while traits control demography. *Front. Earth Sci.* **7**, 10.3389/feart.2019.00083 (2019).

[44] McDowell NG (2011). The interdependence of mechanisms underlying climate-driven vegetation mortality. Trends Ecol. Evol..

[45] Malhi Y (2002). An international network to monitor the structure, composition and dynamics of Amazonian forests (RAINFOR). J. Veg. Sci..

[46] Lopez-Gonzalez, G., Lewis, S. L., Burkitt, M., Baker, T. R. & Phillips, O. L. *ForestPlots.net Database*. http://www.forestplots.net/ (2009).

[47] Lopez-Gonzalez G, Lewis SL, Burkitt M, Phillips OL (2011). ForestPlots.net: a web application and research tool to manage and analyse tropical forest plot data. J. Veg. Sci..

[48] Phillips, O. et al. *RAINFOR Field Manual for Plot Establishment and Remeasurement* Available at: http://www.rainfor.org/upload/ManualsEnglish/RAINFOR_field_manual_version_2016.pdf (2016).

[49] Kohyama, T. S., Kohyama, T. I. & Sheil, D. Definition and estimation of vital rates from repeated censuses: choices, comparisons and bias corrections focusing on trees. *Methods Ecol. Evol.*10.1111/2041-210X.12929 (2017).

[50] Feldpausch TR (2011). Height-diameter allometry of tropical forest trees. Biogeosciences.

[51] R. A language and environment for statistical computing. http://www.R-project.org/ (Vienna, Austria, 2018).

[52] Hothorn T, Bretz F, Westfall P (2008). Simultaneous inference in general parametric models. Biometrical J..

[53] Zanne, A. E. et al. *Data from: Towards a Worldwide Wood Economics Spectrum* (Dryad Data Repository, 2009). 10.1111/j.1461-0248.2009.01285.x 19243406

[54] Fauset, S. et al. Hyperdominance in Amazonian forest carbon cycling. *Nat. Commun.* **6**, 10.1038/ncomms7857 (2015). 10.1038/ncomms7857 PMC4423203 25919449

[55] Flores O, Coomes DA (2011). Estimating the wood density of species for carbon stock assessments. Methods Ecol. Evol..

[56] Collett, D. *Modelling Survival Data in Medical Research* 3 edn (CRC Press, 2015).

[57] Frank E. H. Jr. *rms: Regression Modeling Strategies. v. 5.1-4* (2015).

[58] O’brien RM (2007). A caution regarding rules of thumb for variance inflation factors. Qual. Quant..

[59] Venables, W. N. & Ripley, B. D. *Modern Applied Statistics with S* 4th edn (Springer, 2002).

[60] Burnham, K. P. & Anderson, D. R. *Model Selection and Multimodel Inference: A Practical Information-Theoretic Approach* (Springer, New York, 2002).

[61] Burnham KP, Anderson DR, Huyvaert KP (2011). AIC model selection and multimodel inference in behavioral ecology: some background, observations, and comparisons. Behav. Ecol. Sociobiol..

[62] Terry, M. T. *A Package for Survival Analysis in S* v. 2.38 (Springer, New York, 2015).

