# Author Correction: Tree mode of death and mortality risk factors across Amazon forests

**DOI:** 10.1038/s41467-020-20537-x

**Published:** 2021-01-04

**Authors:** Adriane Esquivel-Muelbert, Oliver L. Phillips, Roel J. W. Brienen, Sophie Fauset, Martin J. P. Sullivan, Timothy R. Baker, Kuo-Jung Chao, Ted R. Feldpausch, Emanuel Gloor, Niro Higuchi, Jeanne Houwing-Duistermaat, Jon Lloyd, Haiyan Liu, Yadvinder Malhi, Beatriz Marimon, Ben Hur Marimon Junior, Abel Monteagudo-Mendoza, Lourens Poorter, Marcos Silveira, Emilio Vilanova Torre, Esteban Alvarez Dávila, Jhon del Aguila Pasquel, Everton Almeida, Patricia Alvarez Loayza, Ana Andrade, Luiz E. O. C. Aragão, Alejandro Araujo-Murakami, Eric Arets, Luzmila Arroyo, Gerardo A. Aymard C., Michel Baisie, Christopher Baraloto, Plínio Barbosa Camargo, Jorcely Barroso, Lilian Blanc, Damien Bonal, Frans Bongers, René Boot, Foster Brown, Benoit Burban, José Luís Camargo, Wendeson Castro, Victor Chama Moscoso, Jerome Chave, James Comiskey, Fernando Cornejo Valverde, Antonio Lola da Costa, Nallaret Davila Cardozo, Anthony Di Fiore, Aurélie Dourdain, Terry Erwin, Gerardo Flores Llampazo, Ima Célia Guimarães Vieira, Rafael Herrera, Eurídice Honorio Coronado, Isau Huamantupa-Chuquimaco, Eliana Jimenez-Rojas, Timothy Killeen, Susan Laurance, William Laurance, Aurora Levesley, Simon L. Lewis, Karina Liana Lisboa Melgaço Ladvocat, Gabriela Lopez-Gonzalez, Thomas Lovejoy, Patrick Meir, Casimiro Mendoza, Paulo Morandi, David Neill, Adriano José Nogueira Lima, Percy Nuñez Vargas, Edmar Almeida de Oliveira, Nadir Pallqui Camacho, Guido Pardo, Julie Peacock, Marielos Peña-Claros, Maria Cristina Peñuela-Mora, Georgia Pickavance, John Pipoly, Nigel Pitman, Adriana Prieto, Thomas A. M. Pugh, Carlos Quesada, Hirma Ramirez-Angulo, Simone Matias de Almeida Reis, Maxime Rejou-Machain, Zorayda Restrepo Correa, Lily Rodriguez Bayona, Agustín Rudas, Rafael Salomão, Julio Serrano, Javier Silva Espejo, Natalino Silva, James Singh, Clement Stahl, Juliana Stropp, Varun Swamy, Joey Talbot, Hans ter Steege, John Terborgh, Raquel Thomas, Marisol Toledo, Armando Torres-Lezama, Luis Valenzuela Gamarra, Geertje van der Heijden, Peter van der Meer, Peter van der Hout, Rodolfo Vasquez Martinez, Simone Aparecida Vieira, Jeanneth Villalobos Cayo, Vincent Vos, Roderick Zagt, Pieter Zuidema, David Galbraith

**Affiliations:** 1 School of Geography, Earth and Enviornmental Sciences, University of Birmingham, Birmingham, UK; 2 School of Geography, University of Leeds, Leeds, UK; 3 Birmingham Institute of Forest Research, University of Birmingham, Birmingham, UK; 4 School of Geography, Earth and Environmental Sciences, University of Plymouth, Plymouth, UK; 5 Department of Natural Sciences, Manchester Metropolitan University, Manchester, UK; 6 International Master Program of Agriculture, National Chung Hsing University, Taichung, Taiwan; 7 Geography, College of Life and Environmental Sciences, University of Exeter, Exeter, UK; 8 Instituto Nacional de Pesquisas da Amazônia, Manaus, Brazil; 9 School of Mathematics, University of Leeds, Leeds, UK; 10 Faculty of Natural Sciences, Department of Life, Imperial College London Sciences, London, UK; 11 Environmental Change Institute, School of Geography and the Environment, University of Oxford, Oxford, UK; 12 UNEMAT – Universidade do Estado de Mato Grosso PPG-Ecologia e Conservação, Campus de Nova Xavantina, Nova Xavantina, MT Brazil; 13 Jardín Botánico de Missouri, Oxapampa, Peru; 14 Forest Ecology and Forest Management Group, Wageningen University and Research, Wageningen, Netherlands; 15 Centro de Ciências Biológicas e da Natureza, Universidade Federal do Acre, Rio Branco, AC Brazil; 16 Instituto de Investigaciones para el Desarrollo Forestal (INDEFOR), Universidad de Los Andes, Mérida, Venezuela; 17 University of California, Berkeley, CA USA; 18 Escuela de Ciencias Agropecuarias y Ambientales, Universidad Nacional Abierta y a Distancia, Boyacá, Colombia; 19 Fundación ConVida, Medellín, Colombia; 20 Instituto de Investigaciones de la Amazonia Peruana, Iquitos, Peru; 21 Instituto de Biodiversidade e Florestas, Universidade Federal do Oeste do Pará, Santarém, Brazil; 22 Center for Tropical Conservation, Nicholas School of the Environment, University in Durham, Durham, NC USA; 23 Projeto Dinâmica Biológica de Fragmentos, Instituto Nacional de Pesquisas da Amazônia Florestais, Manaus, AM Brazil; 24 National Institute for Space Research (INPE), São José dos Campos, SP Brazil; 25 Museo de Historia Natural Noel Kempff Mercado, Universidad Autónoma Gabriel Rene Moreno, Santa Cruz de la Sierra, Bolivia; 26 Wageningen Environmental Research, Wageningen University and Research, Wageningen, Netherlands; 27 Dirección de la Carrera de Biología, Universidad Autónoma Gabriel René Moreno, Santa Cruz de la Sierra, Bolivia; 28 UNELLEZ-Guanare, Herbario Universitario (PORT), Portuguesa, Venezuela Compensation International Progress S.A. Ciprogress–Greenlife, Bogotá, D.C., Colombia; 29 INRAE, UMR EcoFoG, CNRS, Cirad, AgroParisTech, Université des Antilles, Université de Guyane, Kourou, France; 30 Department of Biological Sciences, International Center for Tropical Botany, Florida International University, Miami, FL USA; 31 Centro de Energia Nuclear na Agricultura, Universidade de São Paulo, Piracicaba, Brazil; 32 Universidade Federal do Acre, Campus Floresta, Cruzeiro do Sul, Brazil; 33 UR Forest & Societies, CIRAD, Montpellier, France; 34 Department of Biology, Utrecht, Netherlands; 35 Woods Hole Research Center, Falmouth, MA USA; 36 Laboratório de Botânica e Ecologia Vegetal, Universidade Federal do Acre, Rio Branco, AC Brazil; 37 Laboratoire Evolution et Diversite Biologique, CNRS, Toulouse, France; 38 Inventory and Monitoring Program, National Park Service, Fort Collins, CO USA; 39 Proyecto Castaña, Madre de Dios, Peru; 40 Instituto de Geociências, Faculdade de Meteorologia, Universidade Federal do Para, Belém, Brazil; 41 Department of Anthropology and Primate Molecular Ecology and Evolution Laboratory, University of Texas, Austin, TX USA; 42 National Museum of Natural History, Smithsonian Institute, Washington, DC USA; 43 Universidad Nacional Jorge Basadre de Grohmann, Tacna, Peru; 44 Museu Paraense Emílio Goeldi, Belém, Brazil; 45 Instituto Venezolano de Investigaciones Científicas (IVIC), Caracas, Venezuela; 46 IIAMA, Universitat Politécnica de València, València, Spain; 47 Universidad Nacional de San Antonio Abad del Cusco, Cusco, Peru; 48 Instituto Amazónico de Investigaciones Imani, Universidad Nacional de Colombia Sede Amazonia, Leticia, Colombia; 49 Agteca, Santa Cruz, Bolivia; 50 College of Science and Engineering, James Cook University, Cairns, QLD Australia; 51 Department of Geography, University College London, London, UK; 52 Environmental Science and Policy, George Mason University, Fairfax, VA USA; 53 Research School of Biology, Australian National University, Canberra, ACT Australia; 54 School of Geosciences, University of Edinburgh, Edinburgh, UK; 55 Escuela de Ciencias Forestales, Unidad Académica del Trópico, Universidad Mayor de San Simón, Cochabamba, Bolivia; 56 Facultad de Ingeniería Ambiental, Universidad Estatal Amazónica, Puyo, Ecuador; 57 Universidad Nacional de San Antonio Abad del Cusco, Cusco, Perú; 58 Universidad Autónoma del Beni José Ballivián, Trinidad, Bolivia; 59 Universidad Regional Amazónica Ikiam, Ikiam, Ecuador; 60 Broward County Parks Recreation, Oakland Park, FL USA; 61 Keller Science Action Center, Field Museum, Chicago, IL USA; 62 Instituto de Ciencias Naturales, Universidad Nacional de Colombia, Bogotá, Colombia; 63 Institute of Research for Forestry Development (INDEFOR), Universidad de los Andes, Mérida, Venezuela; 64 Socioecosistemas y Cambio Climatico, Fundacion Con Vida, Medellín, Colombia; 65 Centro de Conservacion, Investigacion y Manejo de Areas Naturales, CIMA Cordillera Azul, Lima, Peru; 66 Universidade Federal Rural da Amazônia, Belém, Brazil; 67 Departamento de Biología, Universidad de La Serena, La Serena, Chile; 68 Guyana Forestry Commission, Georgetown, Guyana; 69 Federal University of Alagoas, Maceió, Brazil; 70 Institute for Conservation Research, Escondido, CA USA; 71 Institute for Transport Studies, University of Leeds, Leeds, UK; 72 Biodiversity Dynamics, Naturalis Biodiversity Center, Leiden, The Netherlands; 73 Systems Ecology, Free University, De Boelelaan 1087, Amsterdam, Netherlands; 74 Department of Biology, University of Florida, Gainesville, FL USA; 75 Iwokrama International Centre for Rainforest Conservation and Development, Georgetown, Guyana; 76 Universidad de los Andes, Mérida, Venezuela; 77 School of Geography, University of Nottingham, Nottingham, UK; 78 Van Hall Larenstein University of Applied Sciences, Leeuwarden, Netherlands; 79 Van der Hoult Forestry Consulting, Leeuwarden, The Netherlands; 80 Núcleo de Estudos e Pesquisas Ambientais – Universidade Estadual de Campinas, Campinas, Brazil; 81 Herbario del Sur de Bolivia, Universidad de San Francisco Xavier de Chuquisaca, Sucre, Bolivia; 82 Tropenbos International, Wageningen, Netherlands

**Keywords:** Forest ecology, Tropical ecology, Forest ecology, Tropical ecology

Correction to: *Nature Communications* 10.1038/s41467-020-18996-3, published online 9 November 2020.

The original version of this Article contained an error in Table 2, where the number of individuals in the “All Amazonia” row was reported as 11,6431 instead of 116,431. Also, the original version of this Article contained an error in the Methods, where the *R*^2^ for the proportion of broken/uprooted dead trees increase per year was reported as 0.12, the correct value being 0.06.

The original version of this Article contained errors in the author affiliations. The affiliation of Gerardo A. Aymard C. with UNELLEZ-Guanare, Herbario Universitario (PORT), Portuguesa, Venezuela Compensation International Progress S.A. Ciprogress–Greenlife, Bogotá, D.C., Colombia was inadvertently omitted. Gerardo A. Aymard C. was incorrectly associated with Instituto de Investigaciones de la Amazonia Peruana, Iquitos, Peru.

This has now been corrected in both the PDF and HTML versions of the Article.

