# Supplementary material for: Tree mode of death and mortality risk factors across Amazon forests
Source: Nat Commun. 2020 Nov 9;11:5515. doi: 10.1038/s41467-020-18996-3 (PMC7652827; doi:10.1038/s41467-020-18996-3)
Supplement: Supplementary file 1 — Supplementary Information [file 41467_2020_18996_MOESM1_ESM.pdf]

Supplementary information

Mode of death and mortality risk factors in Amazon trees

Esquivel-Muelbert *et al.*

Supplementary tables

Table S1 Percentage of different mode of death for different Amazonian regions. The 95% confidence intervals are shown in brackets.

	Standing	Broken/uprooted	n plots	n trees
All Amazon	48 (45-52)	51 (48-54)	125	13862
Northern Amazonia	51 (42-59)	49 (41-57)	18	3049
East Central Amazonia	61 (58-86)	39 (14-43)	10	183
Western Amazonia	44 (28-47)	55 (52-71)	76	8482
Southern Amazonia	55 (49-72)	44 (28-51)	21	2148

Table S2 Number and proportion of trees and taxa with missing traits at species, genus and family levels within our dataset.

		species	genus	family
Mean growth rate	trees	19037 (16%)	7819 (7%)	3722 (3%)
	taxa	2579 (69%)	266 (40%)	30 (26%)
Maximum size	trees	16268 (14%)	7238 (6%)	3663 (3%)
	taxa	2320 (62%)	215 (32%)	25 (22%)
Water deficit affiliation	trees	51798 (44%)	11502 (10%)	3878 (3%)
	taxa	2277 (61%)	191 (29%)	20 (17%)
Wood density	trees	51025 (44%)	9158 (8%)	3630 (3%)
	taxa	2465 (66%)	172 (26%)	9 (8%)

Table S3 Variance inflation factor (VIF) of each of the risk factors included in the models of tree mortality in Amazonia.

	Risk factors	All Amazonia	Northern Amazonia	East-Central Amazonia	Western Amazonia	Southern Amazonia
Stem	D	1.3	1.3	1.3	1.4	1.4
	D^2	1.1	1.1	1.0	1.1	1.0
	rel. growth	1.1	1.1	1.1	1.1	1.1
Species	max D	1.5	1.6	1.6	1.4	1.4
	mean growth	1.7	1.8	1.8	1.7	1.6
	WD	1.3	1.4	1.5	1.3	1.4
	WDA	1.0	1.0	1.0	1.0	1.1

Table S4 Comparison of survival models for standing and broken/uprooted dead trees across Amazonian inventory plots. Models representing the probability of a tree dying standing or being found broken or uprooted, compared using ΔAIC. This analysis was performed using information from 68,593 trees and 11,980 deaths (3,639 standing and 5,409 broken or uprooted) across 116 plots.

Model description	Standing	Broken /uprooted
excluding WDA	0	0
full model	2	2
excluding D^2	23	35
excluding WD	25	15
excluding $(D + D^2)$	38	46
excluding rel. growth	160	19
excluding max D	176	306
excluding individual-level characteristics	202	490
excluding mean growth	383	2
excluding species traits	624	706

Table S5 Risk factors of the best models describing broken/uprooted and standing dead Amazonian trees. Coefficients and standard error, in brackets, and χ^2 for each risk factor of the best models following model selection (Table S3.1). Coefficients that significantly differ from zero considering α = 0.05 are shown in bold.

Risk factors	Standing		Broken/uprooted	
	Coefficients	χ^2	Coefficients	χ^2
D	**-22(5)**	20	**-16(4)**	15
D^2	**19(3)**	37	**19(3)**	56
relGR	**-0.17(0.01)**	135	**-0.04(0.01)**	17
maxD	**-2 x10^{-3}(1 x10^{-4})**	159	**-2 x10^{-3}(1 x10^{-4})**	275
mean growth	**0.2(0.01)**	475	**0.2(0.01)**	511
WD	**-0.7(0.1)**	26	**-0.4(0.1)**	15

Supplementary figures

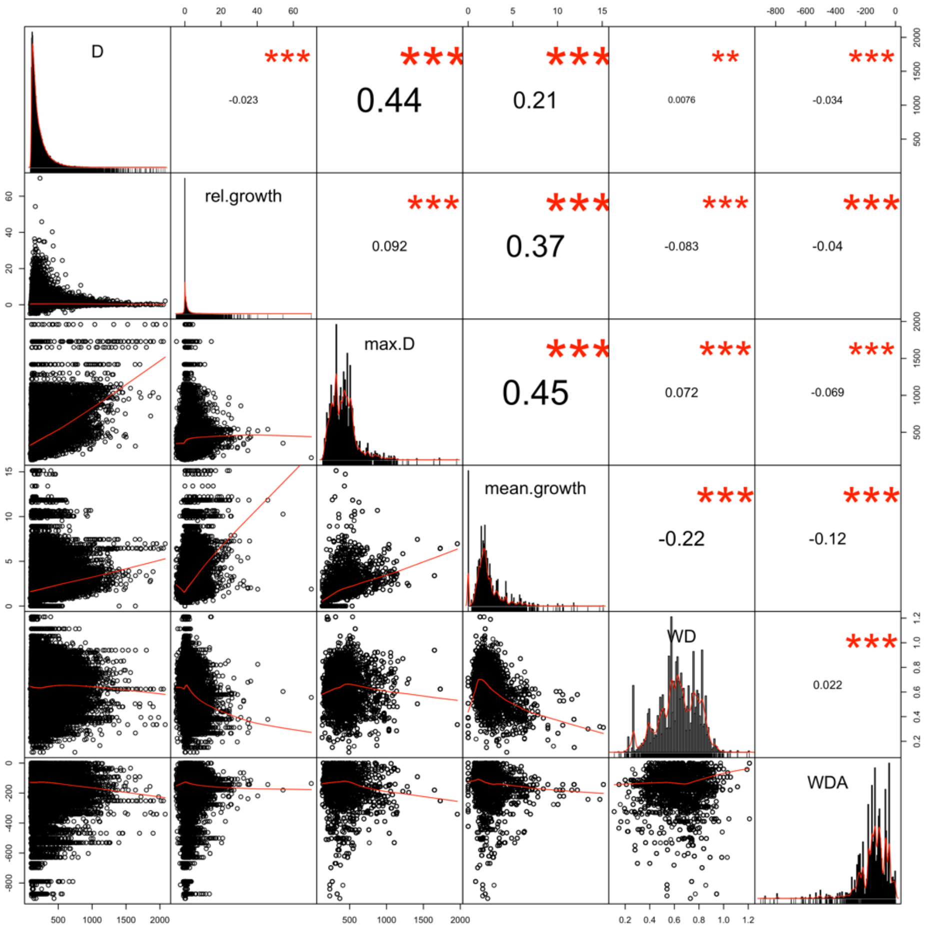

Figure S1 Relationship between predictor variables used in the Cox proportional hazard model. Here we show characteristics of individual trees: size, represented by tree diameter (D) and relative stem diameter growth rates (rel. growth); and species traits: maximum stem diameter size (max D), mean stem diameter growth rate (mean growth), wood density (WD) and drought tolerance represented as water deficit affiliation[1] (WDA). The Kendall τ coefficient of correlation is shown in the top-right panels, font size reflects the value of the correlations. Source data are provided as a Source Data file.

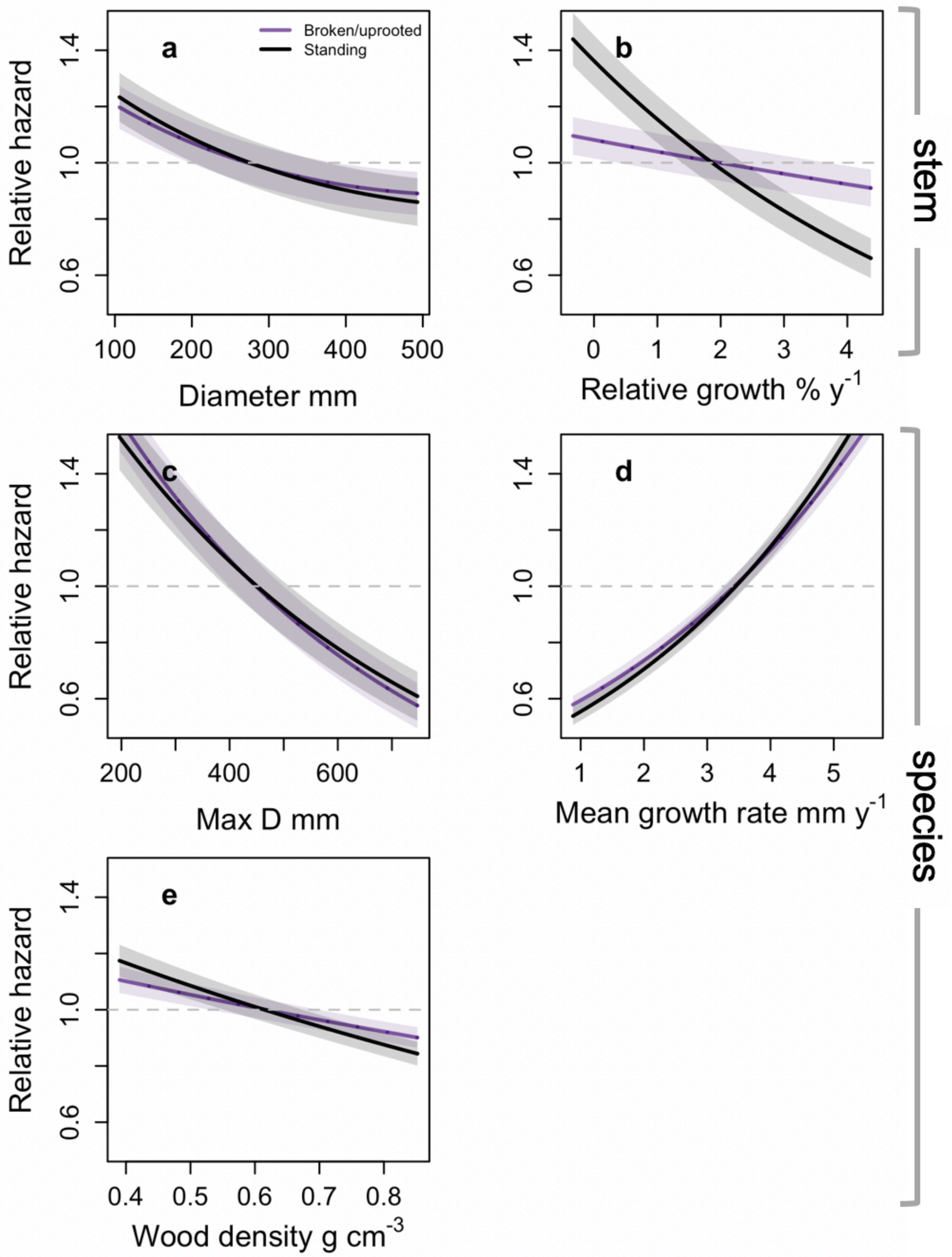

Figure S2 Risk factors associated with death by breakage/uprooting (in purple) and standing death (black) for Amazonian trees. Cox proportional hazard model outputs for the risk factors associated with characteristics of individual trees (**a** size and **b** growth rates) and species traits (**c** maximum size, **d** mean growth rate and **e** wood density). Shaded area represents the standard error for each coefficient. This analysis was performed using information from 68,593 trees and 11,980 deaths (3,639 standing and 5,409 broken or uprooted) across 116 plots. Source data are provided as a Source Data file.

Supplementary References

1 Esquivel-Muelbert, A. *et al.* Seasonal drought limits tree species across the Neotropics. *Ecography* **40**, 618-629, doi:10.1111/ecog.01904 (2017).
