# Supplementary material for: Tree mode of death and mortality risk factors across Amazon forests
Source: Nat Commun. 2020 Nov 9;11:5515. doi: 10.1038/s41467-020-18996-3 (PMC7652827; doi:10.1038/s41467-020-18996-3)
Supplement: Supplementary file 2 — Reporting Summary [file 41467_2020_18996_MOESM2_ESM.pdf]

nature research

Corresponding author(s): Adriane Esquivel Muelbert
Last updated by author(s): Jul 13, 2020

Reporting Summary

Nature Research wishes to improve the reproducibility of the work that we publish. This form provides structure for consistency and transparency in reporting. For further information on Nature Research policies, see Authors & Referees and the Editorial Policy Checklist .

Statistics

For all statistical analyses, confirm that the following items are present in the figure legend, table legend, main text, or Methods section.

n/a	Confirmed	
☐	☒	The exact sample size (*n*) for each experimental group/condition, given as a discrete number and unit of measurement
☐	☒	A statement on whether measurements were taken from distinct samples or whether the same sample was measured repeatedly
☐	☒	The statistical test(s) used AND whether they are one- or two-sided<br>*Only common tests should be described solely by name; describe more complex techniques in the Methods section.*
☐	☒	A description of all covariates tested
☐	☒	A description of any assumptions or corrections, such as tests of normality and adjustment for multiple comparisons
☐	☒	A full description of the statistical parameters including central tendency (e.g. means) or other basic estimates (e.g. regression coefficient) AND variation (e.g. standard deviation) or associated estimates of uncertainty (e.g. confidence intervals)
☐	☒	For null hypothesis testing, the test statistic (e.g. *F*, *t*, *r*) with confidence intervals, effect sizes, degrees of freedom and *P* value noted<br>*Give P values as exact values whenever suitable.*
☒	☐	For Bayesian analysis, information on the choice of priors and Markov chain Monte Carlo settings
☒	☐	For hierarchical and complex designs, identification of the appropriate level for tests and full reporting of outcomes
☒	☐	Estimates of effect sizes (e.g. Cohen's *d*, Pearson's *r*), indicating how they were calculated

Our web collection on statistics for biologists contains articles on many of the points above.

Software and code

Policy information about availability of computer code

Data collection	No software was used for data collection.
Data analysis	All calculations were performed using the R statistical platform R v 3.5.2 (R Development Core Team, 2015) and the packages: multcomp v. 1.4-13, rms v. 5.1-4 and survival v. 3.1-12.<br><br>R codes to generate figures are available at: https://github.com/AEMuelbert/AmazonTreeMortality.git

For manuscripts utilizing custom algorithms or software that are central to the research but not yet described in published literature, software must be made available to editors/reviewers. We strongly encourage code deposition in a community repository (e.g. GitHub). See the Nature Research guidelines for submitting code & software for further information.

Data

Policy information about availability of data

All manuscripts must include a data availability statement. This statement should provide the following information, where applicable:
- Accession codes, unique identifiers, or web links for publicly available datasets
- A list of figures that have associated raw data
- A description of any restrictions on data availability

All permanent inventory plot data are bound to data-use restrictions defined on Forestplots.net. Data to reproduce figures and tables of this study are available online as a data package in the ForestPlots.net website.

Field-specific reporting

Please select the one below that is the best fit for your research. If you are not sure, read the appropriate sections before making your selection.

☐ Life sciences ☐ Behavioural & social sciences ☒ Ecological, evolutionary & environmental sciences

For a reference copy of the document with all sections, see nature.com/documents/nr-reporting-summary-flat.pdf

Ecological, evolutionary & environmental sciences study design

All studies must disclose on these points even when the disclosure is negative.

Study description

We provide a large-scale spatial assessment of mode of tree death (Figure 1 and 2) and the risk factors associated with tree death (Figure 3) across structurally intact forests in Amazonia. To do so, we collected, compiled and analysed data from 124,571 trees (of which 23,683 died during the monitoring period), from 189 forest plots monitored for over 30 years.

Using a Cox proportional hazard approach, we distinguished between the influence of individual condition (size and growth prior to death) and species traits (species mean growth rate, maximum stem diameter size, wood density and drought tolerance, estimated as water deficit affiliation) as risk factors of tree death.

Research sample

We analyzed 124,571 trees from 189 forest plots monitored for over 30 years. All plots analysed were located in lowland (<1,000 m.a.s.l.), terra firme, intact forest and were monitored regularly. The plot average size is 1.23 hectares (95% CI = 1.1, 1.37) with a total area of 331.05 hectares.

Sampling strategy

No sample size calculation was performed. We selected all available plots meeting the criteria described above. All Amazon regions are adequately represented.

Data collection

Forest plots data were collected by teams led by at least one of researchers co-authoring this manuscript across the Amazon basin as part of the RAINFOR network (Malhi et al. 2002), accessed via the ForestPlots.net repository (Lopez-Gonzalez et al. 2009) and following a standardized protocol (Lopez-Gonzalez et al. 2011).

Forest plot data: All trees and palms that have stem diameter at 1.3 m (or above buttresses) of ≥10 cm are measured, tagged, and identified, when possible, to the species level. In every census, when the plot is revisited, the living trees are measured, the new recruits that attain stem diameter ≥10 cm are tagged and measured, and notes are taken about the dead trees. Lianas and nonwoody arborescent individuals from the families Strelitziaceae and Cyatheaceae were excluded from these analyses.

Mode of death: Dead trees were diagnosed as having died standing or non-standing (broken or uprooted) following a standardized protocol for assessing the mode of death based on an analysis of the tree when it is found dead (Phillips et al. 2016, Chao et al. 2009).

Species traits (wood density, maximum size, mean growth and climate affiliation) were obtained from previous studies. Wood density data (in g cm-3) were obtained using previous studies from measurements in different areas of the Amazon (Zanne et al. 2009). Water-deficit affiliation (WDA, in mm) was derived in a previous study using relative abundances across 513 inventory plots distributed along a large water-deficit gradient across the Western Neotropics (Esquivel-Muelbert et al. 2017). Mean growth (in mm y-1) and maximum stem diameter size (in mm) were estimated by previous studies based on a large number of inventory plots distributed across Amazonia (Coelho de Souza 2016, Esquivel-Muelbert 2019). The maximum size represents the 95th quantile of the distribution of size and growth rates across all individuals of a given species (Coelho de Souza 2016, Esquivel-Muelbert 2019).

References:

Malhi, Y. et al. An international network to monitor the structure, composition and dynamics of Amazonian forests (RAINFOR). J. Veg. Sci. 13, 439-450, doi:10.1111/j.1654-1103.2002.tb02068.x (2002).

Lopez-Gonzalez, G., Lewis, S. L., Burkitt, M., Baker, T. R. & Phillips, O. L. ForestPlots.net Database, <http://www.forestplots.net/> (2009).

Lopez-Gonzalez, G., Lewis, S. L., Burkitt, M. & Phillips, O. L. ForestPlots.net: a web application and research tool to manage and analyse tropical forest plot data. J. Veg. Sci. 22, 610-613, doi:10.1111/j.1654-1103.2011.01312.x (2011).

Phillips, O. et al. RAINFOR field manual for plot establishment and remeasurement. (2016).
Chao, K. J., Phillips, O. L., Monteagudo, A., Torres-Lezama, A. & Martinez, R. V. How do trees die? Mode of death in northern Amazonia. J. Veg. Sci. 20, 260-268, doi:10.1111/j.1654-1103.2009.05755.x (2009).

Zanne, A. E. et al. Data from: Towards a worldwide wood economics spectrum (Dryad Data Repository, 2009).
Esquivel-Muelbert, A. et al. Seasonal drought limits tree species across the Neotropics. Ecography 40, 618-629, doi:10.1111/ecog.01904 (2017).

Coelho de Souza, F. et al. Evolutionary heritage influences Amazon tree ecology. Proceedings of the Royal Society B: Biological Sciences 283, doi:10.1098/rspb.2016.1587 (2016).

Esquivel-Muelbert, A. et al. Compositional response of Amazon forests to climate change. Global Change Biology 25, 39-56, doi:doi:10.1111/gcb.14413 (2019).

Timing and spatial scale

Forest dynamics data were collected in monitoring plots of on average 1.23 hectares (95% CI = 1.1, 1.37) distributed across the whole Amazon basin (189 plots, Figure 1). These plots were each measured at least twice for the analysis on the distribution of mortality rates and at least three times for survival analyses (157 plots, Figure 3). Plots were monitored between 1985 and 2015; the average interval between censuses was 2.8 years (95% CI = 2.7, 2.9).

Data exclusions

The size of the plot and the time between census interval may influence the mortality rates. Therefore, we did not include in the analyses plots in which the difference between census intervals was greater than 10 years. Plots smaller than 0.5 hectare were excluded, else joined together when less than 1 km apart.
The 75 individuals (0.06% of the total number of individuals) with relative growth rate more negative than -5%/y were excluded from the analyses, as such negative stem growth is not biologically possible and likely to be a measurement error.
These exclusion criteria were established before the performance of the analyses.

Reproducibility

Our analysis does not include experimental findings. These represent historical observations from across the Amazon region which can never be repeated.

Randomization

We used the Cox proportional hazard model, which estimates the influence of risk factors on the time-to-event response. We consider plot as a random effect (z), or frailty factor, as trees are nested within plots and this factor allows us to account for plot characteristics, e.g. number of censuses, edaphic and climatic conditions.

Basin-level and region-level mortality rates were estimated as the bootstrapped mean and 95% CI of the mortality rates weighted by the area of the plot calculated from 10,000 weighted means of randomly resampled values of plot-level mortality rates across all plots.

Blinding

Blinding was not applied the data analyses.

Did the study involve field work? ☒ Yes ☐ No

Field work, collection and transport

Field conditions

All plots are located in Amazonian forests receiving at least 1,000 mm rainfall annually and with a mean annual temperature of at least 20 °C.

Location

All plots analysed were located in lowland (<1,000 m.a.s.l.), terra firme, intact forest. Location of the 189 plots can be found in Figure 1.

Access and import/export

This manuscript is an outcome of efforts researchers across Amazonia as well as the sustained support of rural communities and local institutions and their funding agencies that make the RAINFOR network possible. A full list of partner institutions (excluding those in the co-author affiliations) can be found in the acknowledgments. Each plot-census represents several months of preparation, transport, data collection, digitalisation and data quality assessment.

We follow the national laws of each individual country to undertake the field work. This work did not required importing or exporting any material.

Disturbance

No significant disturbance was caused by our measurements. Trees were tagged using a single aluminum nail (no iron), avoiding damage to trees due to corrosion.

Reporting for specific materials, systems and methods

We require information from authors about some types of materials, experimental systems and methods used in many studies. Here, indicate whether each material, system or method listed is relevant to your study. If you are not sure if a list item applies to your research, read the appropriate section before selecting a response.

Materials & experimental systems

n/a	Involved in the study
☒	☐ Antibodies
☒	☐ Eukaryotic cell lines
☒	☐ Palaeontology
☒	☐ Animals and other organisms
☒	☐ Human research participants
☒	☐ Clinical data

Methods

n/a	Involved in the study
☒	☐ ChIP-seq
☒	☐ Flow cytometry
☒	☐ MRI-based neuroimaging
